# Pathological manifestation of human endogenous retrovirus K in frontotemporal dementia

**DOI:** 10.1038/s43856-021-00060-w

**Published:** 2021-12-09

**Authors:** Katherine Phan, Ying He, YuHong Fu, Nicolas Dzamko, Surabhi Bhatia, Julian Gold, Dominic Rowe, Yazi D. Ke, Lars M. Ittner, John R. Hodges, Olivier Piguet, Matthew C. Kiernan, Glenda M. Halliday, Woojin Scott Kim

**Affiliations:** 1 The University of Sydney, Brain and Mind Centre, Sydney, NSW Australia; 2 The University of Sydney, School of Medical Sciences, Sydney, NSW Australia; 3 The University of Sydney, The Albion Centre, Sydney, NSW Australia; 4 Faculty of Medicine, Health and Human Sciences, Macquarie University, Sydney, Australia; 5 Dementia Research Centre and Department of Biomedical Sciences, Macquarie University, Sydney, NSW Australia; 6 The University of Sydney, School of Psychology, Sydney, NSW Australia; 7 Institute of Clinical Neurosciences, Royal Prince Alfred Hospital, Sydney, NSW Australia

**Keywords:** Virology, Diseases of the nervous system

## Abstract

**Background:**

Behavioral variant frontotemporal dementia (bvFTD) is a common form of younger-onset dementia with a proportion of cases overlapping pathologically and genetically with amyotrophic lateral sclerosis (ALS). Previous studies have identified that the human endogenous retrovirus K (HERV-K) is elevated in ALS serum and is associated with ALS TDP-43 pathology. In contrast, little is known about HERV-K changes in bvFTD. Here, we investigated the possible role of HERV-K in bvFTD.

**Methods:**

We measured the HERV-K *env* gene in sporadic bvFTD (*N* = 63), sporadic ALS (*N* = 89), and control (*N* = 21) serum by ddPCR. We also analyzed HERV-K *env*, by qPCR, and the HERV-K reverse transcriptase protein, by confocal immunofluorescence microscopy, in the disease-affected superior frontal cortex of bvFTD with TDP-43 pathology.

**Results:**

Here, we show that HERV-K *env* levels are significantly elevated (*P* = 3.5 × 10^−6^) in bvFTD compared to control serum, differentiating cases with an AUC value of 0.867. HERV-K *env* levels are also specifically elevated in the superior frontal cortex of bvFTD with TDP-43 pathology, with the HERV-K reverse transcriptase protein and TDP-43 deposit localized to the neuronal cytoplasm. Furthermore, in a neuronal cell line overexpression of TDP-43 induces HERV-K *env* transcription.

**Conclusions:**

These results suggest that manifestation of HERV-K is associated with bvFTD TDP-43 pathology. Analysis of HERV-K in bvFTD may provide insight into an unrecognized but targetable perturbed pathology.

## Plain language summary

Dementia is a brain disorder characterized by the progressive loss of cognitive abilities. Frontotemporal dementia usually manifests in younger ages compared to other forms of dementia, and sometimes the disease characteristics, such as accumulation of deposits of a protein called TDP-43, resemble those of amyotrophic lateral sclerosis (ALS), a progressive nervous system disease characterized by loss of motor control. Human Endogenous Retrovirus K (HERV-K), a virus that has integrated the human genetic material, has been found to be elevated in patients with ALS with TDP-43 deposits in the nervous system. Here, we measured the level of HERV-K in the blood and brain tissue of patients with frontotemporal dementia and found that it was elevated in both, and this was associated with presence of TDP-43 deposits in the brain. These results improve our understanding of the disease characteristics of frontotemporal dementia.

## Abstract (web summary)

Phan et al. assess the presence of human endogenous retrovirus K (HERV-K) in the serum and brain samples of individuals with behavioral variant frontotemporal dementia (bvFTD) with TDP-43 pathology, as seen in amyotrophic lateral sclerosis. The authors show HERV-K is associated with manifestations of bvFTD with TDP-43 pathology.

## Introduction

Frontotemporal dementia (FTD) is a common form of younger-onset dementia, of which the most common clinical syndrome is the behavioral variant (bvFTD). bvFTD is characterized by disinhibited behavior, loss of emotional processing, and impaired executive function^1–4^. Amyotrophic lateral sclerosis (ALS; also known as motor neuron disease) is a related neurodegenerative disease characterized by loss of motor neurons leading to physical motor impairment^5–7^. bvFTD and ALS exist on a disease spectrum with the majority of cases having the same causal pathogenic protein, trans-activation response (TAR) DNA-binding protein 43 (TDP-43), encoded by *TARDBP*^8–10^. TDP-43 is a DNA/RNA-binding protein that regulates a number of transcriptional and translational processes^11^. It is present in diverse brain cells including neurons. Abnormal deposits of TDP-43 are present in >50% of bvFTD patients and most of ALS patients^12^. Mutations in *TARDBP* are associated with sporadic and familial ALS^13^ and in rare cases, bvFTD^14^.

Up to 15% of bvFTD cases go on to develop ALS symptoms and vice versa^15,16^, and a small subset of cases present with both behavioral and physical symptoms^17^. Currently there are no definitive fluid-based biomarkers for the TDP-43 pathology in these two diseases, although a number of proteins are currently being assessed, including TDP-43^18,19^. Recently, the human endogenous retrovirus K (HERV-K) has been considered as a candidate biomarker for ALS^20–22^. HERV-K is a retrovirus that belongs to the *Beta-retrovirus* genus within the *Retroviridae* family^23,24^. As a retrovirus, it converts its RNA genome into DNA using its reverse transcriptase *pol* gene; the viral DNA then integrates into the human genome at multiple sites. Once integrated, the viral genes are largely inactive or dormant, but can be activated under certain conditions^25,26^. Activation or anomalous expression of HERV-K has long been associated with ALS pathology^21,22,27^. HERV-K DNA, proteins and reverse transcriptase activity have all been shown to be elevated in either or both serum and CSF of ALS patients compared to healthy controls^28,29^. There is also evidence that the human immunodeficiency virus (HIV), which is also a retrovirus belonging to the same *Retroviridae* family, is associated with the activation of HERV-K^30^ and that antiretroviral drug therapy reduce ALS symptoms in HIV patients with ALS^30–32^.

However, despite the fact that bvFTD pathogenesis overlaps with that of ALS, little is known about HERV-K in bvFTD. We therefore hypothesized that HERV-K is implicated in bvFTD TDP-43 pathology. We measured HERV-K in bvFTD, ALS and control serum side-by-side, and generated ROC curves for biomarker development. We analyzed HERV-K expression in multiple regions of bvFTD brain with TDP-43 pathology and determined its cellular localization. We also investigated the role of TDP-43 in HERV-K transcription in a neuronal cell line. We found that HERV-K levels are elevated in both serum and brain tissue in bvFTD compared to controls, and that TDP-43 induces HERV-K expression in a neuronal cell line.

## Methods

### Human serum

Individuals diagnosed with sporadic bvFTD (*N* = 63), sporadic ALS (*N* = 89) and healthy controls (*N* = 21) were recruited from FRONTIER, the FTD clinical research group at the University of Sydney Brain and Mind Centre, from the ForeFront FTD and motor neuron disease clinic at the University of Sydney Brain and Mind Centre, and from a panel of healthy study volunteers^33^ with no neurological or psychiatric disorders, notably no evidence of cognitive impairment. Blood samples were taken at two time points (i.e. visit 1 and visit 2) for each individuals. The mean of number of months between the two visits were FTD 28, ALS 9 and controls 22 months. The study was approved by the University of New South Wales (approval number: HC12573) and the University of Sydney (approval numbers: 2012/160, 2014/539, 2017/928) human research ethics committees. All methods were carried out in accordance with the relevant guidelines and regulations. Blood samples were obtained following written informed consent from the participant and/or primary carer as legal representative. All patients underwent a neurological examination, a comprehensive cognitive assessment and structural brain MRI, and met current consensus diagnostic criteria for bvFTD^34^, ALS^35^ or no neurological disease. Blood samples (9 mL) were collected in tubes (BD Vacutainer SST II Advance Tube #367958), and serum prepared by centrifugation at 3500 rpm for 10 min at 4 °C, which was then aliquoted and stored at −80 °C until use.

### Human brain tissues

Fresh-frozen post-mortem brain tissue samples were obtained with consent from the Sydney Brain Bank and NSW Brain Tissue Resource Centre. All brain donors underwent standardized assessments in life and standardized neuropathological examination, and met current consensus diagnostic criteria for either bvFTD with TDP-43 pathology^36,37^ or no significant neuropathology (controls)^38,39^. Brain tissue samples were obtained following written informed consent from the participant and/or primary carer as legal representative. Consent to publish demographic information in Table 1 has been obtained. Samples from the SFC and cerebellum from 10 bvFTD cases with TDP-43 pathology and 11 controls were used in this study (Table 1). The mean age of the two groups were 72.9 ± 13.0 and 79.5 ± 12.1 years, respectively. Ethics approval for the study of brain tissue was from the University of New South Wales Human Research Ethics (approval number: HC15789).

**Table 1:** FTD patient and control demographic and pathology information of brain tissue samples.

Case/Control	ID	Age	Sex	PMI (h)	Cause of death	Disease durat. (yr)	Brain pathol.	McKenzie type
FTD	1	66	M	39	Cardiorespiratory failure	2	TDP-43	B
FTD	2	62	M	15	Cardiorespiratory failure	3	TDP-43	B
FTD	3	72	F	25	Aspiration pneumonia	1	TDP-43	B
FTD	4	61	M	37	Cardiorespiratory failure	2.5	TDP-43	A
FTD	5	65	F	22	Bronchopneumonia	5	TDP-43	B
FTD	6	84	F	17	Cardiorespiratory failure	8	TDP-43	A
FTD	7	60	M	28	Cardiorespiratory failure	3	TDP-43	A
FTD	8	99	F	13	Cerebral thrombosis	14	TDP-43	A
FTD	9	86	F	25	Cardiorespiratory failure	8	TDP-43	C
FTD	10	74	M	20	Cardiorespiratory failure	7	TDP-43	A
Control	11	85	F	23	Pneumonia	N/A	N/A	N/A
Control	12	79	M	8	Pulmonary embolism	N/A	N/A	N/A
Control	13	89	F	23	Metastatic adenocarcinoma	N/A	N/A	N/A
Control	14	101	F	9	Cardiorespiratory failure	N/A	N/A	N/A
Control	15	84	M	9	Pancreatic cancer	N/A	N/A	N/A
Control	16	93	F	15	Gastrointestinal bleeding	N/A	N/A	N/A
Control	17	74	M	10	Respiratory failure	N/A	N/A	N/A
Control	18	63	M	24	Cardiac failure	N/A	N/A	N/A
Control	19	66	M	23	Cardiac failure	N/A	N/A	N/A
Control	20	74	F	20	Cancer	N/A	N/A	N/A
Control	21	67	F	15	Cancer	N/A	N/A	N/A

### HERV-K and HERV-W ddPCR assay

Serum HERV-K and HERV-W cell-free and genomic DNA levels were assessed using ddPCR protocol established by Avindra Nath (NIH, Bethesda, MD, USA) and as previously described^32^. 300 µL serum were centrifuged for 10 min to remove cellular debris and total nucleic acids extracted using the QIAmp Viral RNA Mini Kit (Qiagen, Australia) following the manufacturer’s instructions. Nucleic acids were not treated with DNAse as HERV-K viral particles contain both RNA and DNA^40–42^. 2.5 µL extracted nucleic acids were added to a reaction mix that consisted of 12.5 µL ddPCR Supermix (no dUTP) (BioRad, Australia), 1.25 µL HERV *env* primer (900 nm) and probe (250 nm) mix, 1.25 µL ddPCR RPP30 copy number assay and 7.5 µL nuclease-free water. Droplets were generated on a QX100 Droplet Generator (BioRad, Australia) and PCR performed on a BioRad C1000 Thermal Cycler (BioRad, Australia) according to the following program: 10 min at 95 °C, 40 cycles of 30 s at 95 °C and 1 min at 60 °C, and 10 min at 95 °C. Droplets were assessed in a QX200 Droplet Digital PCR System (Bio-Rad, Australia). HERV-K and HERV-W levels were recorded as the ratio of HERV DNA copies to RPP30 DNA copies. Primer and probe sequences were as follows: HERV-K Fwd: 5′ ATTTGGTGCCAGGAACTGAG 3′; HERV-K Rev: 5′ GCTGTCTCTTCGGAGCTGTT 3′ and HERV-K Probe: 5′ 6-FAM-AGGAGTTGCTGATGGCCTCG-Iowa Black FQ 3′ (BioRad, Australia), HERV-W Fwd: 5′ GTATGTCTGATGGGGGTGGAG 3′, HERV-W Rev: 5′ CTAGTCCTTTGTAGGGGCTAGAG 3′, HERV-W Probe: 5′ HEX/ZEN-TCCCAACTGACCCAGGTACATAGC-Iowa Black FQ 3′ (IDT, Singapore). RPP30 copy number assay Hex labeled (dHsaCP2500350, BioRad, Australia) was used in the HERV-K assay. RPP30 copy number assay FAM labeled (dHsaCP2500313, BioRad Australia) was used in the HERV-W assay.

### RNA extraction and quantitative PCR

RNA was isolated from brain tissues (20 mg) using TRIzol reagent (Invitrogen) following the manufacturer’s protocol. All procedures were carried out using RNase-free reagents and consumables. Four microgram of RNA was reverse transcribed into cDNA using Moloney-murine leukemia virus (M-MLV) reverse transcriptase and random primers (Promega, Madison, Wisconsin, USA) in 20 μl reaction volume. HERV-K and HERV-W *env* levels were measured by quantitative polymerase chain reaction (qPCR) using the BioRad CFX Connect (BioRad, Australia) and the fluorescent dye SYBR Green (Bio-Rad), following the manufacturer’s protocol. Briefly, each reaction (20 μl) contained 1× mastermix, 5 pmoles of primers and 1 μl of cDNA template. Amplification was carried out with 40 cycles of 94 °C for 15 s and 60 °C for 1 min. Gene expression was normalized to the geometric mean of three housekeeper genes, GAPDH, β-actin, and PPIA. A no-template control was included for each PCR amplification assay. The primer sequences were –

HERV-K: ATTTGGTGCCAGGAACTGAG and GCTGTCTCTTCGGAGCTGTT;

HERV-W: GTATGTCTGATGGGGGTGGAG and CTAGTCCTTTGTAGGGGCTAGAG;

GAPDH: AATGAAGGGGTCATTGATGG and AAGGTGAAGGTCGGAGTCAA;

β-actin: GAATTCTGGCCACGGCTGCTTCCAGCT and AAGCTTTTTCGTGGATGCCACAGGACT; and

PPIA: AGGGTTCCTGCTTTCACAGA and GTCTTGGCAGTGCAGATGAA.

### Immunohistochemistry

Formalin-fixed, paraffin-embedded sections (10 µm) from superior frontal cortex (SFC) were deparaffinized in xylene and rehydrated through graded ethanol, followed by antigen retrieval with citrate buffer (pH 6.0) using a pressure cooker (Aptum Bio Retriever 2100, Aptum Biologics Ltd, UK) at a peak temperature of ~121 °C and gradually cooling to room temperature. Endogenous peroxidase was blocked with 1% hydrogen peroxide in 50% ethanol. Sections were blocked with 5% normal horse serum, then incubated with HERV-K RT antibody (Abnova, H00002087-A01, mouse, 1:250) at 4 °C for two overnights, followed by the secondary antibody ImmPRESS™-AP Anti-Mouse IgG Polymer Detection Kit (Abacus-ALS, VEMP-5402) as per manufacturer’s instructions. They were then counterstained with hematoxylin and cover-slipped. Negative controls (without primary antibody or secondary antibody) were performed for each immunohistochemistry run, and no signals were detected.

### Immunofluorescence

Formalin-fixed, paraffin-embedded sections (10 µm) from SFC were deparaffinized in xylene and rehydrated through graded ethanol, followed by antigen retrieval with citrate buffer (pH 6.0) using a pressure cooker (Aptum Bio Retriever 2100, Aptum Biologics Ltd, UK) at a peak temperature of ~121 °C and gradually cooling to room temperature. Endogenous peroxidase was blocked with 1% hydrogen peroxide in 50% ethanol. Sections were first labeled with HERV-K RT antibody (Abnova, H00002087-A01, mouse, 1:250), followed by the secondary antibody ImmPRESS™-HRP Horse Anti-Mouse IgG (HRP) Polymer (Abacus-ALS, VEMP-5402) at room temperature for 30 min prior to applying FluorTM 488 Tyramide Reagent (Thermo Fisher, B40953). Sections were then stripped using citrate buffer (pH 6.0) and pressure cooker prior to blocking with 2.5% donkey serum and 1% BSA. The primary incubation was conducted with a cocktail containing TDP-43 antibody (Proteintech, 10782-2-AP, rabbit, 1:400) and NeuN antibody (Biolegend, SIG-39860, mouse IgG2b, 1:100) at 4 °C for two overnights. Sections were then washed with PBS and incubated with the corresponding secondary antibodies (ThermoFisher Scientific, A-10042 and A-31571, 1:250) and 4′,6-diamidino-2-phenylindole DAPI (Sigma-Aldrich, D9542, 1 mg/ml) at room temperature for 2 h. Next, the slides were treated with 70% Sudan Black for 30 min and 10 mM CuSO_4_ in 50 mM ammonium acetate buffer (pH 5.0) for 1 h to quench auto-fluorescence signals prior to cover-slipping with anti-fade fluorescence mounting medium (DAKO, S3023) and then sealed with nail polish. Negative controls (without primary antibodies or secondary antibodies) were performed for each immunohistochemistry run, and no signals were detected in each case.

### Microscopy imaging

For immunohistochemistry, stained sections were scanned using an Olympus VS120 Slide Scanner with the same focus and exposure settings. For immunofluorescence, multiple sections were examined and representative images were captured with a Nikon C2 confocal microscope and associated Nikon NIS Elements software (version 4.60). Images were adjusted for contrast and converted to TIFF format on Fiji software (ImageJ version 2.0.0-rc-69/1.52p).

### Cell culturing

SHSY-5Y neuronal cells overexpressing the human wild type *TARDBP* gene and the vector-only control^43^ were cultured in 12-well plates in Dulbecco’s modified Eagle’s medium (DMEM) containing 10% fetal calf serum, 1% Glutamax, 0.5% glucose, 100 IU/ml penicillin and 100 μg/ml streptomycin at 37 °C in humidified air containing 5% CO_2_. After 48 h the cells were harvested and total RNA prepared for gene expression studies.

### Statistical analysis

Statistical analyses were performed using SPSS Statistics software 26 (IBM, Chicago, IL, USA). For comparisons between bvFTD, ALS, and control groups, univariate analysis (general linear model), with age and gender as covariates, was used and significance set at *P* < 0.05. Receiver operating characteristic (ROC) analysis was used to determine the best cut-off for HERV-K and curves generated in SPSS 26. All other graphs were generated using GraphPad Prism 7.

## Results

### Validation of results from an antiretroviral clinical trial

**Fig. 1:**
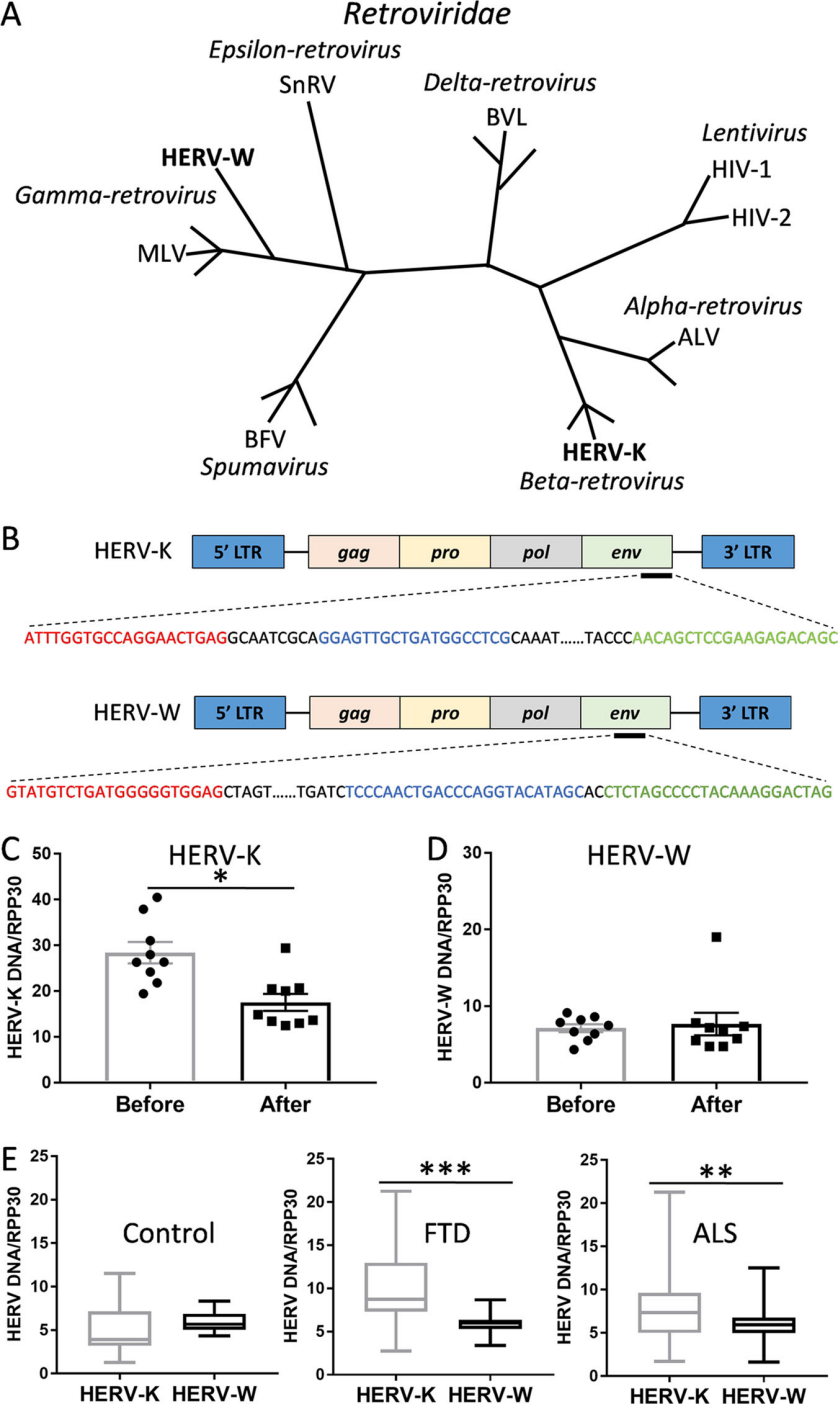
Validation of HERV-K measurements of a clinical trial data. **A** Phylogeny of *Retroviridae* family with HERV-K and HERV-W belong to different genera (italicized) (adapted from Weiss et al. ^24^). **B** ddPCR amplification of the HERV-K and HERV-W *env* gene with forward primer (red), reverse primer (green) and probe (blue). **C** Validation of the antiretroviral drug Triumeq effect on HERV-K levels before and after 24 weeks of treatment in ALS patients (*n* = 9). Data represent individual patients, mean and SE as error bars. **D** HERV-W levels before and after 24 weeks of treatment with Triumeq in ALS patients (*n* = 9). Data represent individual patients, mean and SE as error bars. **E** A comparison of HERV-K and HERV-W *env* levels in control (*n* = 21), FTD (*n* = 63) and ALS (*n* = 89) serum. Data represent median with min and max as whiskers. *P < 0.05, **P < 0.001, ***P < 0.0001.

HERV-K is pathologically associated with ALS and has been shown to be elevated in ALS serum^28,29^. We measured HERV-K levels in serum collected from ALS patients (*N* = 9) that were treated with the antiretroviral drug Triumeq in a Phase I clinical trial^32^. We also measured HERV-W, which is phylogenetically different to HERV-K^24^ (Fig. 1A) and is not known to be associated with ALS (and therefore could serve as a negative internal control). The aim was to validate the assay (Supplementary Results, Table S1 and Fig. S1) and clinical data using the digital droplet PCR (ddPCR) methodology that quantifies the HERV-K *env* gene in serum (Fig. 1B). Our assay confirmed the significant decreases in HERV-K levels following the 24-week drug treatment (Fig. 1C). The HERV-W levels were not altered with the drug treatment (Fig. 1D). Once we validated the published data, we proceeded with testing our new cohorts using the same ddPCR strategy. We detected both HERV-K and HERV-W in all the neurological samples, and that HERV-K was present at higher levels compared to HERV-W in both bvFTD and ALS, but not in control serum (Fig. 1E).

### Investigating HERV-K as a diagnostic biomarker for bvFTD

**Fig. 2:**
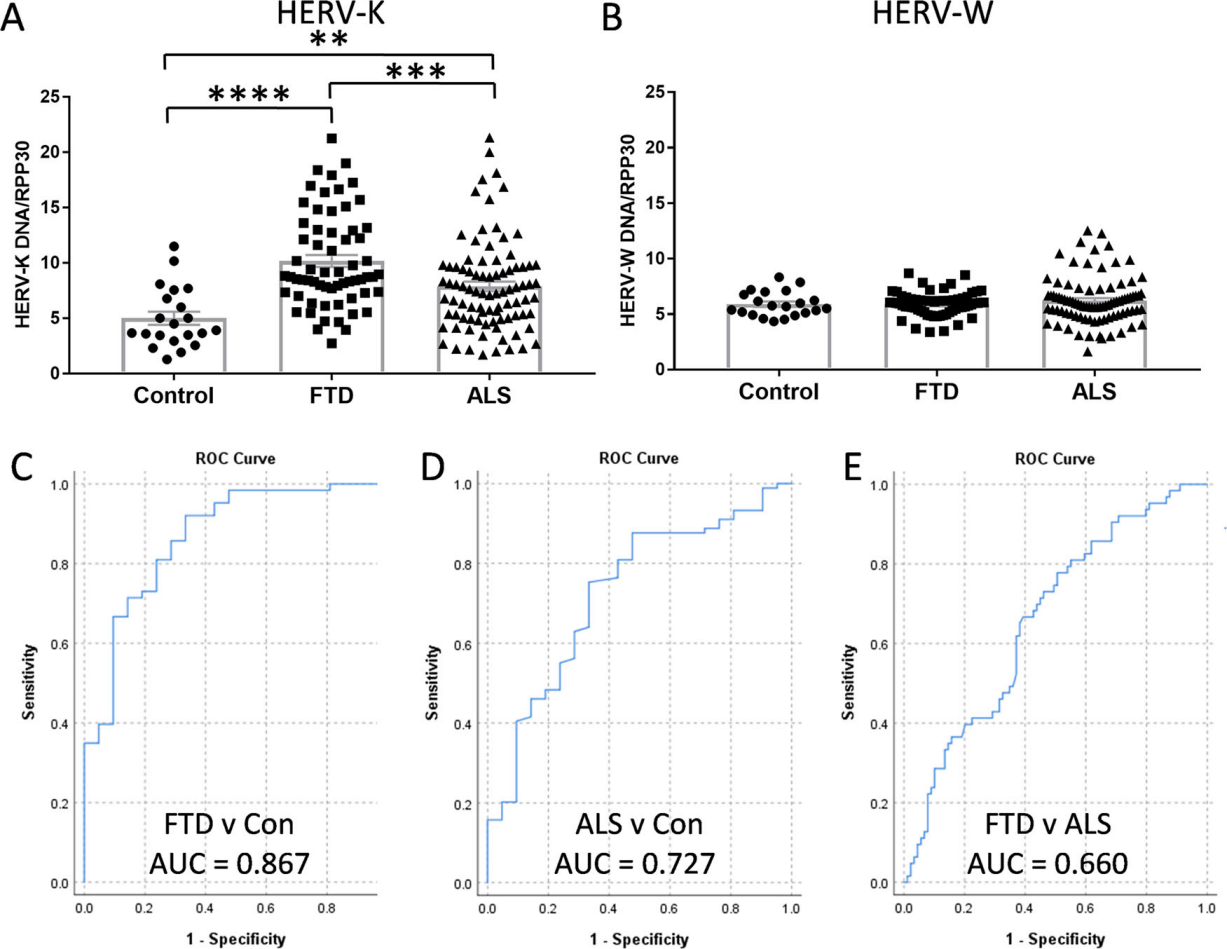
A comparison of HERV-K levels in FTD and ALS. **A** HERV-K *env* levels in FTD (*n* = 63), ALS (*n* = 89) and control (*n* = 21) serum as measured by ddPCR. **B** HERV-W *env* levels in FTD (*n* = 63), ALS (*n* = 89) and control (*n* = 21) serum as measured by ddPCR. (C) HERV-K ROC curve for FTD vs. Con. **D** HERV-K ROC curve for ALS vs. Con. **E** HERV-K ROC curve for FTD vs. ALS. Data represent individual patients, mean and SE as error bars, **P < 0.01, ***P < 0.001, ****P < 0.000005.

The aim of this study was two-fold, first, to determine whether HERV-K levels were altered in bvFTD, and second, to explore whether HERV-K could be developed as a diagnostic tool for bvFTD. We measured the HERV-K and HERV-W *env* gene in sporadic bvFTD (*N* = 63), sporadic ALS (*N* = 89), and control (*N* = 21) serum side-by-side (Supplementary Data 1). HERV-K levels were significantly increased in bvFTD compared to controls, as was the case for ALS, as expected (Fig. 2A). Interestingly, HERV-K levels of bvFTD were even higher than those of ALS (Fig. 2A). HERV-W levels were not altered in either bvFTD or ALS (Fig. 2B). We then generated ROC curves and found that HERV-K could discriminate bvFTD from controls (AUC = 0.867) at a HERV-K level of 5.18 (92% sensitivity, 67% specificity) (Fig. 2C), and ALS from controls (AUC = 0.727) at a HERV-K level of 5.02 (75% sensitivity, 67% specificity) (Fig. 2D). The AUC value between bvFTD and ALS was 0.660 (Fig. 2E).

### HERV-K and bvFTD TDP-43 pathology

**Fig. 3:**
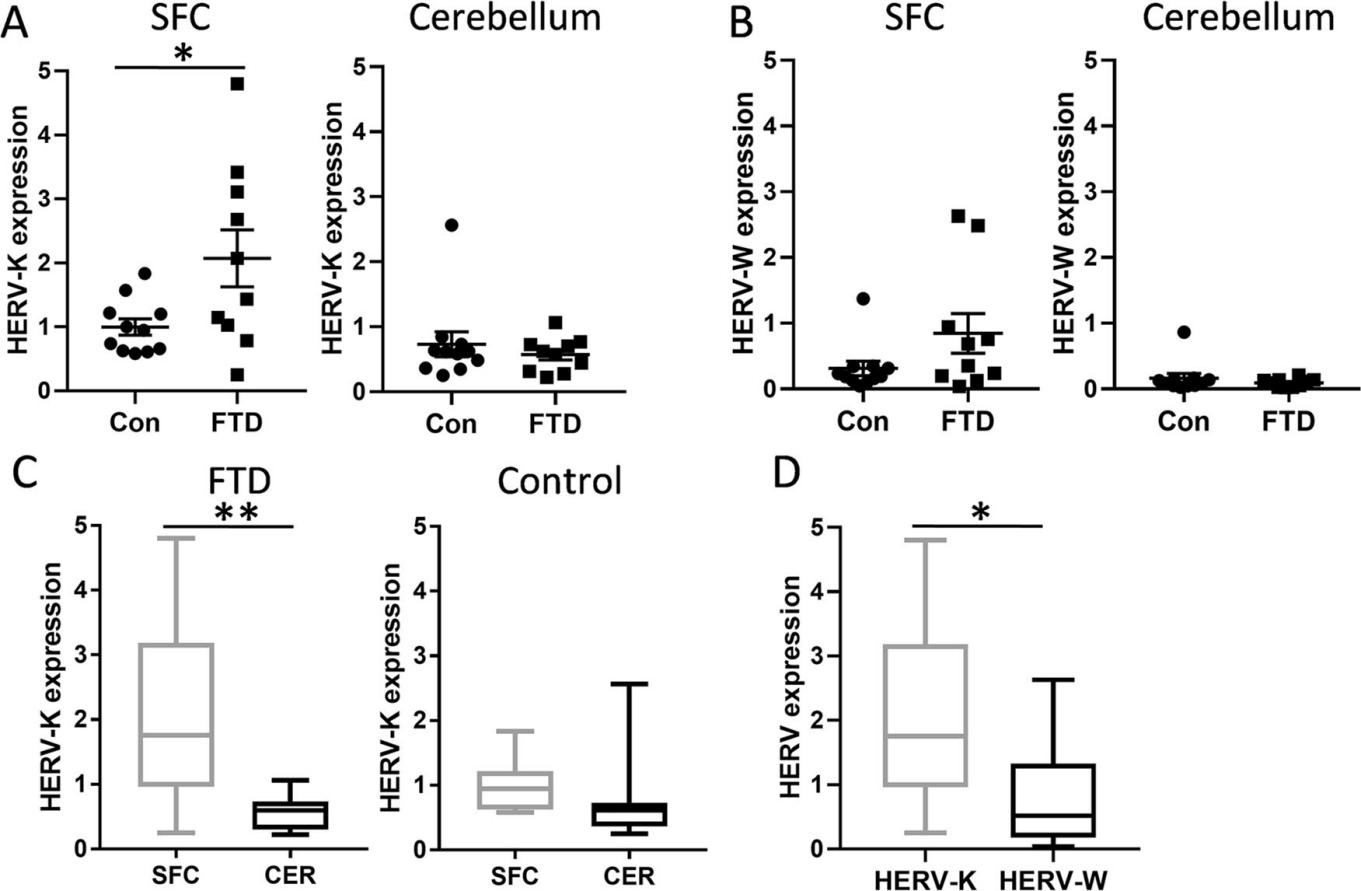
Expression of HERV-K in FTD brain. **A** HERV-K *env* mRNA expression in the superior frontal cortex (SFC) and cerebellum of FTD (*n* = 10) and control (*n* = 11) brain as measured by qPCR. Data represent mean and SE as error bars. **B** HERV-W *env* mRNA expression in the SFC and cerebellum of FTD (*n* = 10) and control (*n* = 11) brain as measured by qPCR. Data represent mean and SE as error bars. **C** A comparison of HERV-K *env* expression in the SFC and cerebellum in FTD (*n* = 10 SFC, *n* = 10 cerebellum) and control (*n* = 11 SFC, *n* = 11 cerebellum). Data represent median with min and max as whiskers. **D** A comparison of HERV-K and HERV-W *env* expression in FTD SFC (*n* = 10). Data represent median with min and max as whiskers. *P < 0.05, **P < 0.01.

To further explore HERV-K as a possible marker for bvFTD TDP-43 pathology and to understand the link between serum and brain HERV-K levels, we measured the HERV-K *env* transcript expression in bvFTD with TDP-43 pathology and control brain (Table 1) in the SFC, a brain region affected by FTD, and the cerebellum, a brain region largely unaffected by FTD pathologies. The HERV-K *env* transcript expression was significantly elevated only in the SFC in bvFTD with TDP-43 pathology compared to controls (Fig. 3A). We also measured HERV-W *env* transcript expression in the same samples and found that it was not altered in either SFC or cerebellum (Fig. 3B). The HERV-K *env* transcript expression was significantly higher in SFC compared to cerebellum only in bvFTD with TDP-43 pathology (Fig. 3C). Furthermore, the HERV-K *env* transcript expression was significantly higher than the HERV-W *env* transcript expression in the SFC of bvFTD with TDP-43 pathology (Fig. 3D), as was the case in bvFTD serum (Fig. 1E). These results suggest that HERV-K is specifically associated with bvFTD TDP-43 pathology and that HERV-K could serve as a diagnostic marker for this pathology.

### TDP-43 induces HERV-K *env* transcription

**Fig. 4:**
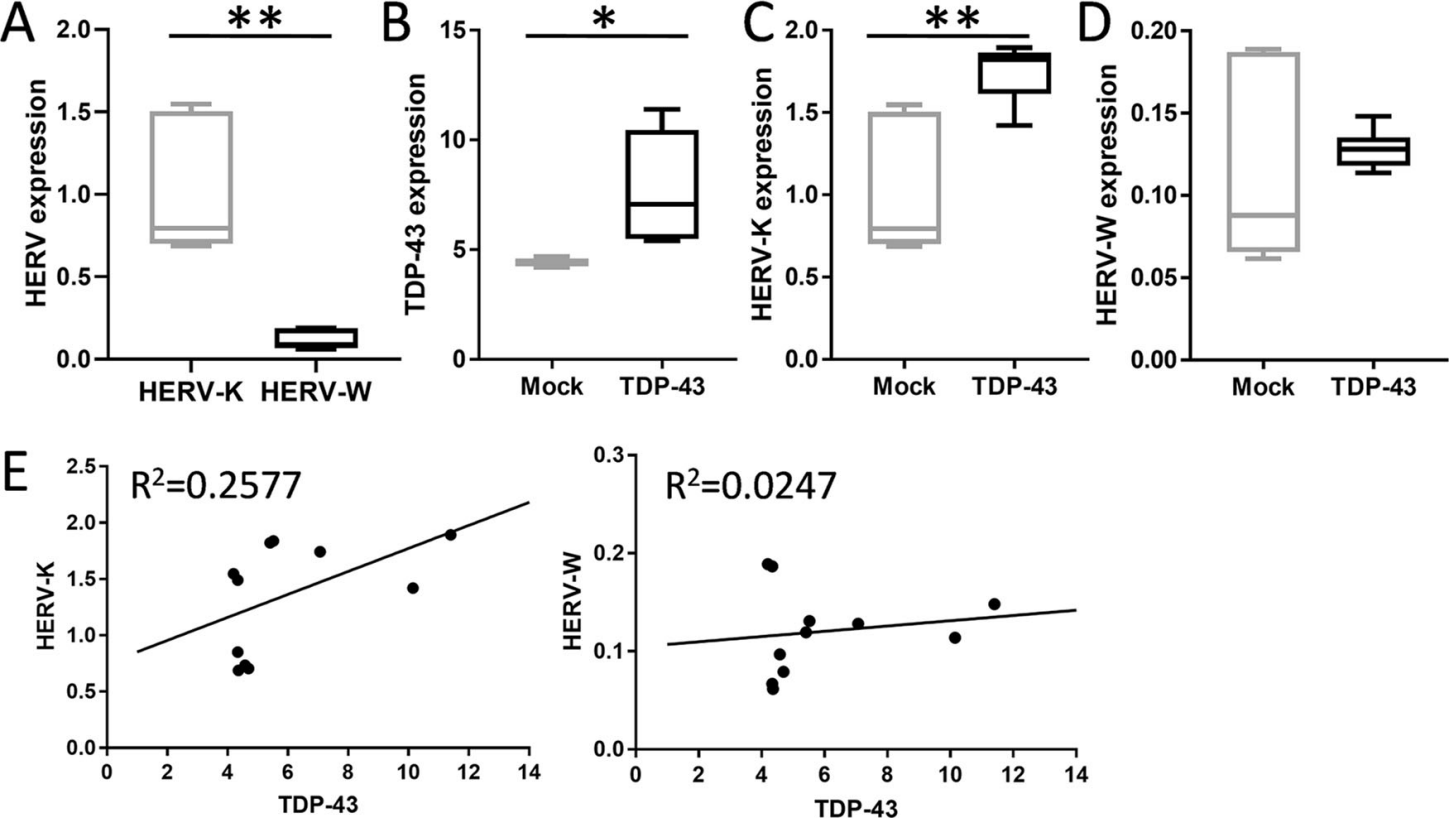
TDP-43 induces HERV-K transcription. **A** HERV-K and HERV-W *env* levels in SH-SY5Y neuronal cells (*n* = 6 biological replicates). **B** Expression of TDP-43 in SH-SY5Y neuronal cells transfected with the human wild type *TARDBP* cDNA (TDP-43) (*n* = 5 biological replicates) or empty vector plasmid control (mock) (*n* = 6 biological replicates). **C** Analysis of impact of TDP-43 on HERV-K *env* transcription in TDP-43 cells (*n* = 5 biological replicates) and mock cells (*n* = 6 biological replicates). **D** Analysis of impact of TDP-43 on HERV-W *env* transcription in TDP-43 cells (*n* = 5 biological replicates) and mock cells (*n* = 6 biological replicates). Boxplot data represent median with min and max as whiskers. *P < 0.05, **P < 0.005. **E** Correlation analysis of TDP-43 to HERV-K and HERV-W *env* transcription (*n* = 11 individual samples).

Since TDP-43 is intrinsically linked to both bvFTD and ALS TDP-43 pathology and the fact that TDP-43 is a known transcriptional factor that binds to the TAR element of members of *Retroviridae* family^22^, we sought to investigate whether TDP-43 regulates HERV-K transcription. Using a SH-SY5Y cell model of bvFTD/ALS that overexpresses the human *TARDBP* gene and an empty-vector control^43^, we assessed the impact of TDP-43 expression on HERV-K *env* transcription. Firstly, we demonstrated that both HERV-K and HERV-W *env* transcripts were expressed in SH-SY5Y neuronal cells and that HERV-K levels were significantly higher than HERV-W levels (Fig. 4A), as was the case in the brain tissues. Secondly, we confirmed the increased TDP-43 expression in the *TARDBP*-overexpressing cells (Fig. 4B). We then showed that TDP-43 expression caused significant increases in HERV-K *env* transcription (Fig. 4C), but not HERV-W *env* transcription (Fig. 4D). Furthermore, TDP-43 expression levels correlated with HERV-K but not HERV-W levels (Fig. 4E). These results strongly corroborate our brain data and indicate that increased TDP-43 expression promotes HERV-K transcription.

### Cellular localization of HERV-K reverse transcriptase in FTD brain

**Fig. 5:**
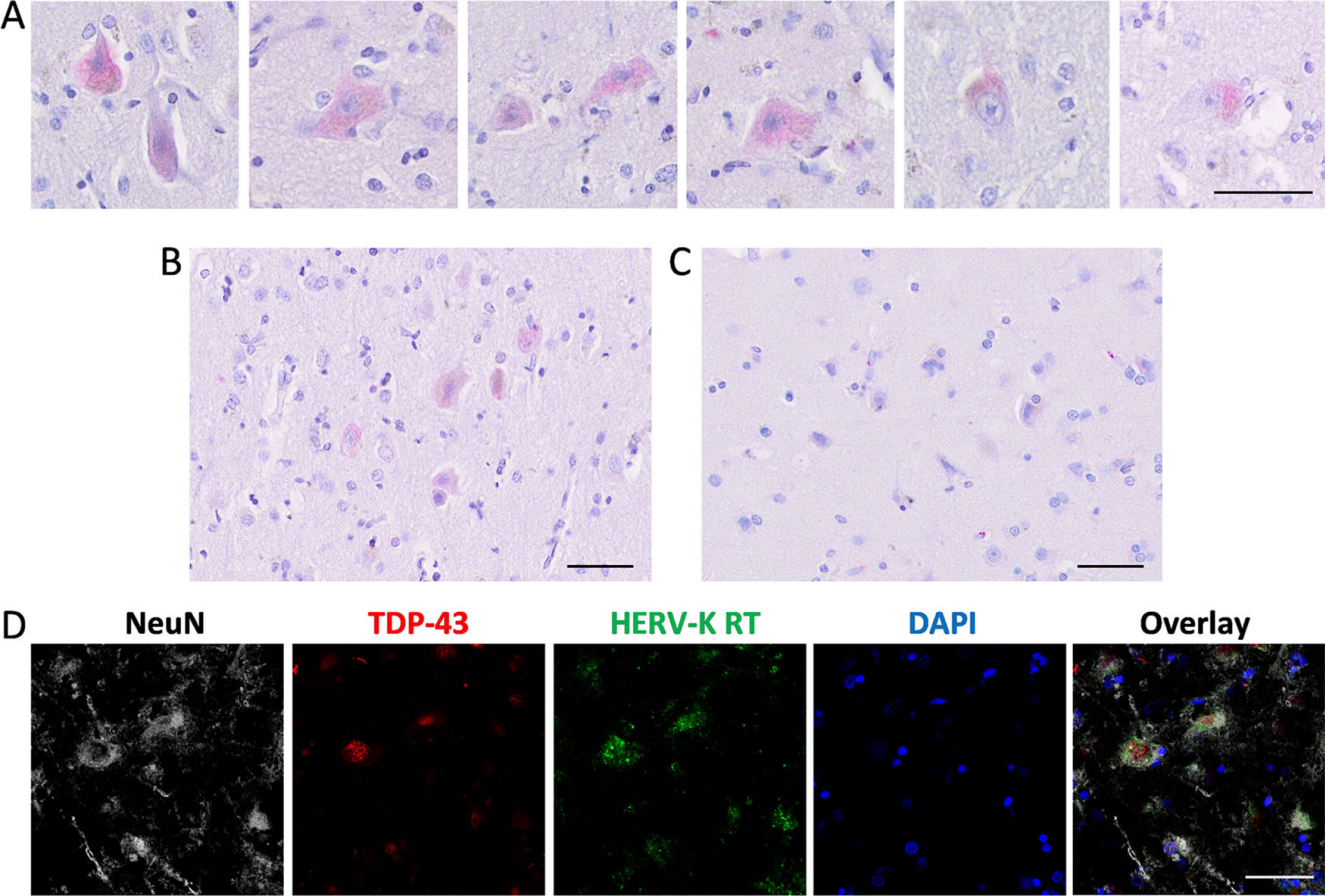
Immunohistochemical analysis of HERV-K reverse transcriptase (RT) in the superior frontal cortex of FTD brain. **A** Staining of HERV-K RT (red) in neurons in FTD SFC tissue. **B** Staining of HERV-K RT in FTD SFC tissue. **C** Staining of HERV-K RT in control SFC tissue. **D** Confocal immunofluorescence of neurons in FTD SFC (case #10) stained with NeuN (gray), TDP-43 (red), HERV-K RT (green) and DAPI (blue). Scale bar = 50 µm.

To further investigate the pathological link between TDP-43 expression and HERV-K transcription in bvFTD brain with TDP-43 pathology, we analyzed the localization of HERV-K reverse transcriptase (RT) in pathologically affected neurons. We prepared formalin-fixed paraffin-embedded tissue sections from the superior frontal cortex (SFC) and stained with HERV-K RT antibody. HERV-K RT was detected in neurons in the FTD cases (Fig. 5A) with a larger field of view (Fig. 5B), along with the staining of control SFC tissue (Fig. 5C). Analysis of neurons with TDP-43 deposits in FTD SFC using confocal immunofluorescence microscopy showed that HERV-K RT, along with TDP-43, localized to the cytoplasm and pathologic nucleus (Fig. 5D).

### A longitudinal analysis of HERV-K in bvFTD and ALS serum

**Fig. 6:**
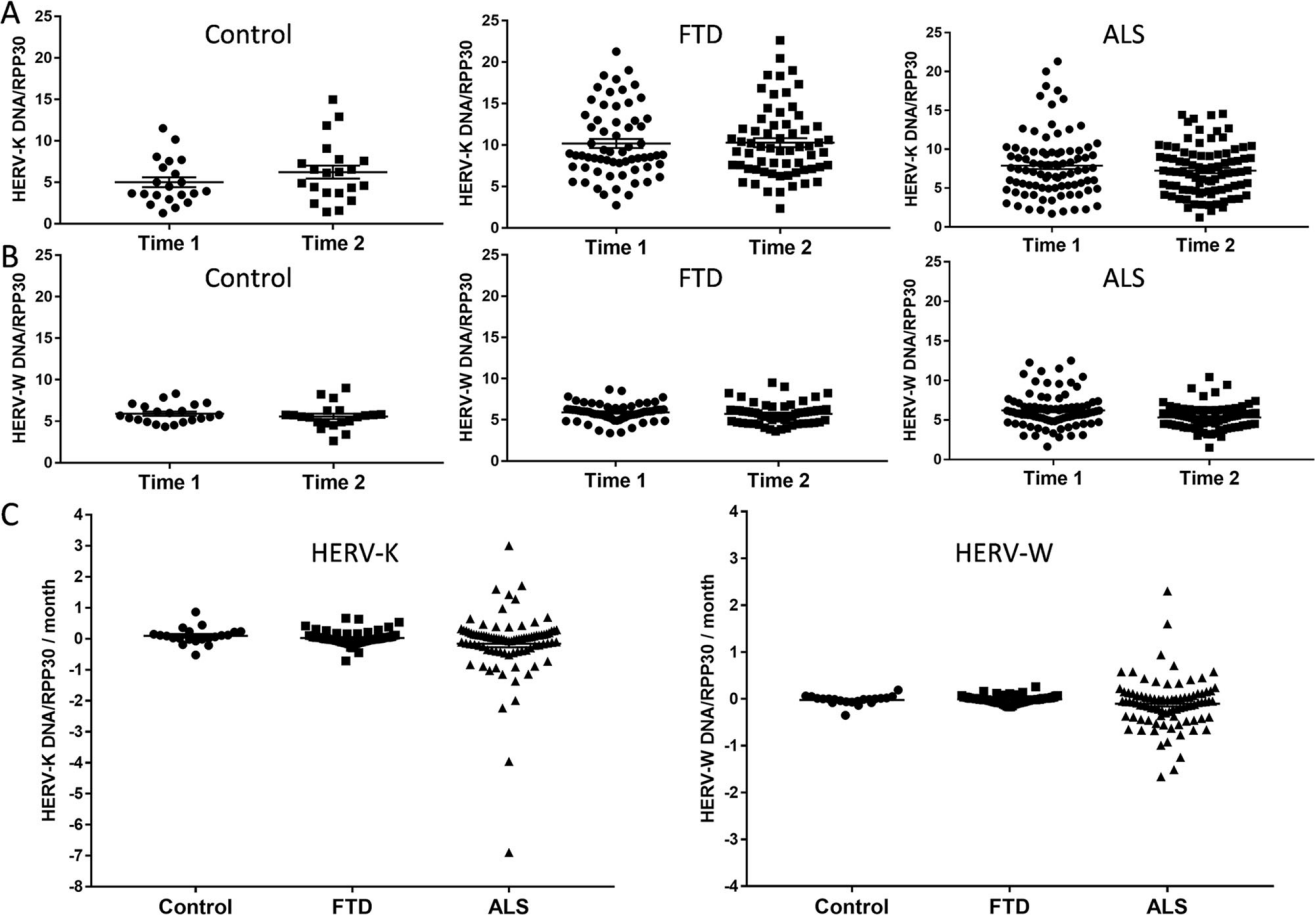
A longitudinal assessment of HERV-K levels in FTD and ALS serum. **A** HERV-K levels at two time points; the mean of number of months between the two time points were 28 for FTD (*n* = 63), 9 for ALS (*n* = 89) and 22 for controls (*n* = 21). **B** HERV-W levels at two time points in FTD (*n* = 63), ALS (*n* = 89) and control (*n* = 21) serum. **C** Rate of change in HERV-K and HERV-W levels in FTD (*n* = 63), ALS (*n* = 89) and control (*n* = 21) serum. Data represent individual patients, mean and SE as error bars.

We were also interested in whether HERV-K levels change with disease progression. Blood samples were taken at two time points (i.e. visit 1 and visit 2) for each individual. The average number of months between the two visits were 28 for bvFTD, 9 for ALS and 22 for controls (note that ALS is a rapid-progressing disease). We found that HERV-K levels were not altered between the two time points for all three groups (Fig. 6A). Likewise, HERV-W levels were not altered (Fig. 6B). We also measured the rates of change (change per month) and found that they were static for bvFTD and controls, but variable for ALS (Fig. 6C).

## Discussion

The present series of studies investigated the possible role of HERV-K in bvFTD TDP-43 pathology and measured HERV-K levels in bvFTD serum and brain tissues. We used the validated ultrasensitive ddPCR to detect and quantitate HERV-K *env* DNA in bvFTD, ALS and control serum side-by-side. First, we confirmed that HERV-K levels were elevated in ALS serum compared to controls. Importantly, we revealed that HERV-K levels were also elevated in bvFTD serum compared to controls, and that the levels were even higher than those of ALS serum. To understand the link between serum and brain HERV-K levels, we measured HERV-K *env* transcript expression in the SFC (a FTD affected region) and cerebellum (a FTD unaffected region) in bvFTD with TDP-43 pathology and control brain. We found that HERV-K *env* transcript expression was elevated in bvFTD with TDP-43 pathology compared to controls only in the SFC. Furthermore, we demonstrated that TDP-43 pathology promoted HERV-K *env* transcription and that HERV-K RT, along with TDP-43, was localized to the neuronal cytoplasm. These results suggest that HERV-K is associated with bvFTD with TDP-43 pathology, and can be measured in serum, as is the case with ALS.

The significance or ramification of HERV presence in humans is underscored by the fact that retroviral genes have permanently integrated into the human genome and constitute up to 8% of the human genome^44,45^. Although the integrated retroviral genes are largely inactive, they are thought to still retain some of their functions^46,47^, and/or are activated under certain conditions. In ALS, these conditions are, for example, inflammation^48^, binding of TDP-43 to RNA^49^, relaxation of heterochromatin by C9orf72 peptides^50^ and infection of another virus^22,48,51,52^. Consistent with this, HIV, which belongs to the same *Retroviridae* family as HERV-K, appears to exacerbate ALS pathology. Clinical studies have shown that antiretroviral HIV drugs lower HERV-K levels and ALS symptoms^32,53^. HERV-K levels were also shown to be closely associated with the development of motor neuron symptoms in HIV patients^30^.

Of relevance to the present studies, HERV-K transcripts have been detected in cortical and spinal neurons of ALS^27^. In vivo and in vitro studies have revealed that increases in HERV-K expression causes cell toxicity, degeneration of the motor neurons and progressive motor dysfunction, indicating that HERV-K load is associated with neurodegeneration severity^22^. In mouse models of ALS, the expression of HERV-K *env* resulted in decreases in motor cortex volume and synaptic activity in pyramidal neurons, and muscle atrophy and motor dysfunction^22^. Interestingly, the HERV-K RT protein co-localizes with TDP-43 deposits in ALS brain^27^. The abnormal TDP-43 deposits are present in the brain of most of ALS patients and ~50% of bvFTD patients^12^. In a *Drosophila* model of bvFTD expressing human TDP-43, Gypsy, a *Drosophila* endogenous retrovirus, was shown to facilitate TDP-43-mediated propagation of neurodegeneration^54^. Consistent with these observations, our results showed that the HERV-K *env* transcript expression is elevated in the SFC of bvFTD brain with TDP-43 pathology, and that TDP-43 localizes with the RT protein in the cytoplasm of neurons in bvFTD brain. Furthermore, we demonstrated, using a neuronal cell model, that TDP-43 expression induces HERV-K *env* transcription. As stated before, TDP-43 is a transcriptional factor that binds to both DNA and RNA and regulates numerous genes at both transcriptional and translational levels. In neurons, TDP-43 binds to thousands of RNAs^55^. In the case of HERV-K, TDP-43 binds to a promoter sequence in the long terminal repeat upstream of HERV-K^22^. Interestingly, TDP-43 also binds to the TAR element of HIV; the TAR element is essential for viral promoter activation and subsequent virus replication^56^. However, a previous study found that although TDP-43 bound to the HERV-K promoter during inflammation or proteasomal deficiencies, HERV-K transcription was not altered. Instead, HERV-K viral protein accumulated in neurons with overexpression of pathogenic forms of TDP-43^57^. Another study using murine models showed that both overexpression and depletion of TDP-43 upregulated HERV-K expression among other transposable elements^49^. Further adding to the complexity of the relationship between TDP-43 and HERV-K expression, another study found that knockdown of TDP-43 reduced HERV-K expression^22^. Clearly, further research is required to fully understand the exact relationship between TDP-43 and HERV-K expression.

In contrast to HERV-K, HERV-W (a *Retroviridae* negative internal control) levels were consistently unaltered in bvFTD, as well as in ALS. Our results are consistent with previous findings that showed that HERV-W *env* levels are unaltered in ALS serum, and that there is no association between HERV-W levels and ALS^21,58,59^. HERV-W is phylogenetically distant from HERV-K and it could be under different activation pathways or unresponsive to the same mechanisms that regulate HERV-K (HERV-K is phylogenetically closer to HIV). However, in one study, the HERV‐W element encoding syncytin was shown to be elevated in muscle biopsies of ALS patients. However, the authors concluded that this elevation is likely to be due to downstream macrophage response to inflammation rather than a primary pathogenic event in ALS^59^.

In this study, we also examined the potential of HERV-K as a diagnostic tool for bvFTD. We showed that HERV-K is a good discriminator of bvFTD from controls. However, although HERV-K levels were significantly higher in bvFTD compared to ALS serum, the AUC value was not high enough to be a good discriminator of bvFTD from ALS. This is not surprising knowing that there are clinical and pathological overlap in these two diseases. When considering HERV-K as a diagnostic tool for bvFTD, it is also important to recognize that HERV-K levels can also be affected by other conditions or factors. For example, HERV-K levels are elevated with certain cancers^60^, and in healthy people over the age of 45^20^ (although they are more elevated in bvFTD (see the “Results” section)). Research on the possible link between HERV-K and other neurodegenerative diseases is emerging. For example, HERV-K RNA was shown to be detectable more frequently in Alzheimer’s disease (AD) CSF compared to controls, and that there was a distinct correlation of upregulated HERV-K and Toll-like receptor 8 RNA expression in AD brain^61^. However, another study found that there was no difference in serum or CSF HERV-K protein levels in AD compared to controls^21^. Interestingly, other HERVs, such as HERV-Fc1, have been linked to AD through Tau pathology^62^.

In conclusion, we demonstrated that HERV-K is biochemically related to bvFTD with TDP-43 pathology and that HERV-K could potentially serve as a blood biomarker for bvFTD. Though further multicenter studies are required to confirm the association between serum HERV-K levels and to clarify the link between HERV-K and TDP-43 pathology, our study has provided new insights into an unrecognized perturbed pathology in bvFTD and opened a new area of research for understanding the pathogenesis of bvFTD with TDP-43 pathology.

## Supplementary information

Supplementary Information

Description of Additional Supplementary Files

Supplementary Data 1

## Data Availability

Source data for the main figures in the manuscript can be accessed as Supplementary Data 1. The remaining data cannot be made publicly available because the ethical approval and the informed consent from the patients included in this study did not cover placing the data into publicly open repositories. Relevant portions of the data can be accessed from the authors upon relevant ethical approval by contacting the corresponding author on reasonable request.
