# Supplementary material for: Pathological manifestation of human endogenous retrovirus K in frontotemporal dementia
Source: Commun Med (Lond). 2021 Dec 9;1:60. doi: 10.1038/s43856-021-00060-w (PMC8788987; doi:10.1038/s43856-021-00060-w)

Pathological manifestation of human endogenous retrovirus K in frontotemporal dementia

Katherine Phan[1,2#], Ying He[1,2#], YuHong Fu[1,2#], Nicolas Dzamko[1,2], Surabhi Bhatia[1,2], Julian Gold[3], Dominic Rowe[4], Yazi D. Ke[5], Lars M. Ittner[5], John R. Hodges[1], Olivier Piguet[1,6], Matthew C. Kiernan[1,7], Glenda M. Halliday[1,2] and Woojin Scott Kim[1,2*]

Supplementary Results

We assessed the ddPCR method utilized in the Triumeq drug trial for measuring HERV-K levels. In this protocol the HERV-K load measured is a combination of HERV-K DNA from the viral particle, cell-free DNA and gDNA from any residual PBMCs. There is no conversion of the RNA extracted from the viral particle nor is there removal of DNA. As such, we firstly attempted to measure just the HERV-K viral RNA load from serum. In a small trial (8 Con, 8 FTD and 8 ALS), RNA and DNA were extracted from serum as described by Avindra Nath (NIH, Bethesda, MD, USA) in reference #32. Nucleic acid extract was treated with DNAse and the treated sample converted to DNA using SuperScript IV VILO with ezDNAse (Invitrogen, Australia). When ddPCR was performed on these samples, the majority of samples had no detectable HERV-K (Table S1). This result is consistent with previous findings that the lack of signal is likely due to the majority of nucleic acid within the HERV-K viral particle consisting of DNA (references #40-42). Secondly, as this method measures HERV-K from gDNA we also determined whether genomic copies of HERV-K in FTD patients differ from that of controls. qPCR was used to measure relative HERV-K copy number in gDNA extracted from leukocytes (FTD N=26, control N=17). Results showed that there was no significant difference between FTD and controls (Figure S1). Thus, any

differences in HERV-K levels from nucleic acids extracted from serum, would be due to differences in HERV-K viral load.

Table S1. ddPCR HERV-K positive droplet counts from samples treated or untreated with DNAse.

	**HERV-K Positive Droplet Count**	
**Sample**	**Untreated**	**DNAse Treated**
S1	134	2
S2	446	0
S3	493	0
S4	744	0
S5	86	4
S6	987	0
S7	1015	0
S8	1314	16
S9	802	0
S10	73	0
S11	154	0
S12	963	0
S13	3131	0
S14	534	0
S15	503	0
S16	566	0
S17	44	0
S18	358	0
S19	2736	0
S20	462	0
S21	242	0
S22	306	0
S23	786	0
S24	625	0

Fig. S1. Comparison of HERV-K relative copy number in FTD patients and controls.

HERV-K *env* copy number in gDNA extracted from leukocytes as measured by qPCR.

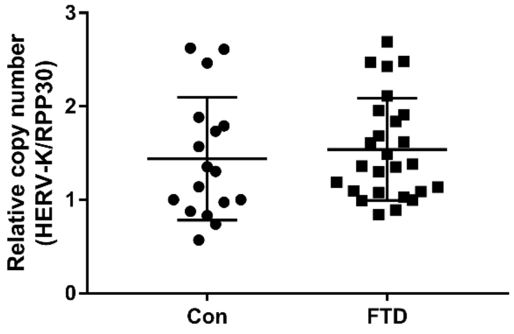

Supplement: Supplementary file 1 — Supplementary Information [file 43856_2021_60_MOESM1_ESM.pdf]
