# Supplementary material for: Pathological manifestation of human endogenous retrovirus K in frontotemporal dementia
Source: Commun Med (Lond). 2021 Dec 9;1:60. doi: 10.1038/s43856-021-00060-w (PMC8788987; doi:10.1038/s43856-021-00060-w)
Supplement: Supplementary file 2 — Description of Additional Supplementary Files [file 43856_2021_60_MOESM2_ESM.pdf]

Description of Additional Supplementary Files

File Name: Supplementary Data 1

Description: HERV-K and HERV-W *env* ddPCR measurements of FTD (n=63), ALS (n=89) and control (n=21) serum (Tab 1). HERV-K and HERV-W *env* qPCR measurements of the superior frontal cortex (SFC) and cerebellum of FTD (n = 10) and control (n = 11) brain (Tab 2). Expression of TDP-43, HERV-K and HERV-W in SH-SY5Y neuronal cells transfected with the human wild type *TARDBP* cDNA (TDP-43) (n = 5) or empty vector plasmid control (mock) (n = 6) (Tab 3).
